# Term alpaca placenta glycosylation profile and its correlation with pregnancy maintenance and fetal survival

**DOI:** 10.3389/fcell.2023.1193468

**Published:** 2023-06-05

**Authors:** Miluska Beatriz Navarrete Zamora, Thamires Santos da Silva, Mônica Duarte da Silva, Gustavo Henrique Doná Rodrigues Almeida, Leandro Norberto da Silva-Júnior, Bianca de Oliveira Horvath-Pereira, Amanda Trindade Baracho Hill, Francisco Acuña, Ana Claudia Oliveira Carreira, Rodrigo da Silva Nunes Barreto, Alberto Sato Sato, Maria Angélica Miglino

**Affiliations:** ^1^ Department of Surgery, School of Veterinary Medicine and Animal Science, University of São Paulo, São Paulo, Brazil; ^2^ Facultad de Medicina Veterinaria, Universidad Nacional Mayor de San Marcos, San Borja, Brazil; ^3^ Facultad de Ciencias Veterinárias, Universidad Nacional de La Plata, Buenos Aires, Brazil; ^4^ Centre of Natural and Human Sciences, Federal University of ABC, Santo André, Brazil

**Keywords:** alpaca, glycobiology, term placenta, extreme environments, cellular communication

## Abstract

Alpaca is a South American camelid, particularly present in Peruvian highlands, where oxygen concentration and atmospheric pressure are very low. Due to this fact, gestational physiology has adapted to preserve the conceptus’ and mother’s health. In this context, several cellular and molecular features play an essential role during and at the end of gestation. Structural carbohydrates act on maternal–fetal communication, recognize exogenous molecules, and contribute to placental barrier selectivity. Therefore, this study aimed to characterize the structural carbohydrate profiles that are present in the term alpaca placenta, kept in their natural habitat of around 4,000 m height. For this propose, 12 term alpaca placentas were collected, and the material was obtained at the time of birth from camelids raised naturally in the Peruvian highlands, in the Cusco region. All placenta samples were processed for histological analysis. A lectin histochemical investigation was performed using 13 biotinylated lectins, allowing us to determine the location of carbohydrates and their intensity on a semi-quantitative scale. Our results demonstrated that during term gestation, the epitheliochorial alpaca placenta shows a high presence of carbohydrates, particularly glucose, α-linked mannose, *N-acetylglucosamine* β (GlcNAc), galactose (αGal), and *N-acetylgalactosamine* α (GalNAc), present in the trophoblast, amnion epithelium, and mesenchyme, as well as the presence of sialic acid residues and low affinity for fucose. In fetal blood capillaries, the presence of bi- and tri-antennary complex structures and α-linked mannose was predominated. In conclusion, we characterized the glycosylation profile in the term alpaca placenta. Based on our data, compared to those reported in the bibliography, we suggest that these carbohydrates could participate in the labor of these animals that survive in Peruvian extreme environments.

## 1 Introduction

Alpaca (*Vicugna pacos*) is the most common camelid species in South America, particularly found in Andean countries such as Peru, Bolivia, Chile, and Argentina. These animals are adapted to high altitudes of 3,600 and 4,900 m and are able to survive and procreate in this extreme environment characterized by low oxygen levels, low atmospheric pressure, and hostile cold temperatures (Olivera et al., 2003a; Jiménez et al., 2010).

Several studies already demonstrated that hypoxic conditions during embryo development and fetal growth are able to cause many deleterious effects in organogenesis, which may lead to sexual, motor, and neurological dysfunctions (Zamudio, 2004). In the cellular microenvironment, low oxygen levels are related to epigenetic alterations, endocrine dysfunctions, mitochondrial oxidative stress, and higher reactive oxygen species (ROS) production, thus leading to cell damage (Zhao et al., 2021). Some studies conducted with ovine placentas comparing the impact of high and low altitudes on placental morphology highlighted that high-altitude conditions have a significant impact on placental development and establishment (Parraguez et al., 2006). The observed placental adaptations are correlated with the improvement of maternal–fetal surface contact, which may attenuate the effects of lower oxygen tensions (Soares et al., 2017). Recently, in humans, a similar effect has been observed in high-altitude population pregnancies, in which placental and uterine physiological adaptations provided a sort of compensation for the hypoxic conditions, allowing normal fetal growth (Moore et al., 2011; Dolma et al., 2022).

Due to their economic importance for the Andean population and the demonstration of a great efficient capacity to convert native vegetation into high-quality meat and fibers (Inigues and Alem, 1996), the reproductive aspects of alpacas have been broadly studied, once they have several morphofunctional adaptations that allow their survival in such conditions. In addition, these animals characterize a main livelihood for many farmers in the central Andean countries of South America (Quispe et al., 2009).

In addition to these socioeconomic aspects, this species has a unique reproductive feature that may represent, based on more specific studies, an interesting animal model to investigate the impact of the environment on gestation, which may be correlated with the same conditions that Andean pregnant women are exposed to (Benirschke and Kaufmann, 1995; Araya et al., 2000; Firshman et al., 2013). Moreover, some studies related to the alpaca placenta and the placentation process have shown that these animals also have epitheliochorial, diffuse placentas with the presence of multinucleated giant cells (Carter and Enders, 2013), which are very similar to those placentas found in pigs, horses, and camels (Olivera et al., 2003b).

In this context, maternal–fetal communication is essential to nourish and protect the embryo for the entire gestation against exogenous agents through the placental barrier (Jones et al., 1997; 2007). Previous studies have indicated that this cellular communication is mediated mainly by a sugar code that assists on the placental selectivity (Gabius et al., 2004; Habermann et al., 2021). Each animal exhibits a singular glycosylation profile, which is characterized by structural carbohydrates such as mannose, glucose, fucose, galactose, *N-acetylgalactosamine*, *N-acetylglucosamine*, and sialic acid (Walsh, 2010; Varki et al., 2015), that plays an important role in intracellular communication overcoming nucleic acids and proteins (Nair and Salomon, 2020). These carbohydrates require a link with a glycoprotein, specifically lectins, that assists on their functionality (Lviii et al., 2004). Therefore, numerous carbohydrates play specific roles in infections, inflammations, immunity, fertilization, gestation, tumor metastasis, and transplacental nutrient transportation (Gabius et al., 2002; Lviii et al., 2004; Varki, 2017).

Some studies involving glycosylation at the maternal and fetal interface camelid placenta were carried out, which demonstrated that maternal and fetal glycotypes presented mutual compatibility, determining the stability of this interface (Jones et al., 2002; 2008). This presence of glycans may make hybrid production and development difficult (Jones et al., 2002; 2008). In addition to this context, the glycosyltransferase expression profile varies in response to several external stimuli, which is regulated by epigenetic mechanisms (Fernández-Ponce et al., 2021; Klasić and Zoldoš, 2021). These results corroborate the fact that environmental conditions may modulate the glycosylation pattern and, thus, membrane glycoprotein functionality, which constitutes an additional level of cellular process regulation (Russo, 2011). An example of such an environmental modulation is that in certain hostile conditions that alpacas are exposed, these animals mobilize their own micronutrients like selenium to support the survival of the conceptus. This transplacental nutrient transportation is highly influenced by the glycosylation pattern, once a modification in this profile may compromise the maternal–fetal communication (Ziganshina et al., 2021).

Therefore, given the roles that carbohydrates play on several biological processes, including placentation and transplacental nutrient transportation, this study aimed to investigate and determine the glycosylation pattern in the term alpaca placenta.

## 2 Materials and method

### 2.1 Ethics committee

This investigation was approved by the Ethics Committee on Animal Use (CEUA) of the Faculty of Veterinary Medicine and Animal Science, University of São Paulo (7213120719).

### 2.2 Sampling

Twelve (n = 12) term alpaca placentas were collected in the Cusco/Peru region of the Peruvian Andes (altitude 4,338 m). The placentas were collected after the labor and birth of the newborns, coming from the registered mother alpacas, and then, the placentas were fixed in 10% formaldehyde solution for later histological and lectin histochemical analyses.

### 2.3 Histology

Random samples were collected from eight parts of the 12 placentas, including the following fetal membranes: chorioallantoic and amnion membranes. Then, the tissue fragments (1–3 cm) were dehydrated in increasing concentrations of ethanol (70%–100%), cleared, embedded in paraffin, and cut using a microtome of 5 µm thickness to be stained with hematoxylin and eosin (H&E) as a standard histological technique, and Masson’s trichrome under light microscopy.

### 2.4 Lectin histochemistry

Sections of the same samples used for the histological study were incubated separately with biotinylated lectins. Batteries of 13 biotinylated lectins were used from Vector Labs^®^, following standardized protocols using the avidin–peroxidase system (Table 1).

**TABLE 1:** Lectins used, with indications on their origin, dilution, and carbohydrate affinity.

Acronym	Lectin	Dilution	Carbohydrate affinity^a^
CON- A	*Canavalia ensiformis*	2 mg/ml	β-D-Man and α-D-Glc
SBA	*Glycine maximus*	2 mg/ml	α-D-GalNAc and β-D-GalNAc
WGA	*Triticum vulgaris*	2 mg/ml	β-D-GlcNAc and NeuNAc
DBA	*Dolichos biflorus*	2 mg/ml	α-D-GalNAc
UEA-I	*Ulex europaeus*	2 mg/ml	L-Fuc
RCA-I	*Ricinus communis*	2 mg/ml	β-Gal
PNA	*Arachis hypogea*	7.5 mg/ml	β-D-Gal (β1-3)> D-GalNAc
GSL-I	*Griffonia simplicifolia lectin I*	2 mg/ml	D-Gal
PSA	*Pisum sativum*	2 mg/ml	α-D-Glc and α-D-Man
LCA	*Lens culinaris agglutinin*	2 mg/ml	α-Man
PHA-E	*Phaseolus vulgaris erythroagglutinin*	2 mg/ml	Complex structures (bisected bi/tri-antennary complex *N-linked sequences*)
PHA-L	*Phaseolus vulgaris leucoagglutinin*	2 mg/ml	Complex structures (non-bisected tri/tetra-antennary complex N-linked sequences)
sWGA	Succinyl-wheat germ agglutinin	2 mg/ml	β1-4-D- GlcNac

^a^ Gal, galactose; GalNAc, *N-acetylgalactosamine*; Glc, glucose; GlcNAc, *N-acetylglucosamine*; L-Fuc, *L-fucose*; Man, mannose; NeuNAc, *N-acetylneuraminic acid* = *sialic acid*.

### 2.5 Procedure

The sections were cleared in a xylene substitute (Neo Clear ^®^) and rehydrated in ethanol (100%). Endogenous peroxidase blockade was performed for 30 min in methanol mixed with 10 volume of hydrogen peroxide (3%). Rehydration continued with the decreasing concentration of ethanol (96%, 70%, and 50%). Subsequently, phosphate-buffered saline (PBS) was used for washing. Then, the samples were incubated in bovine serum albumin (BSA 1%)to avoid unspecific unions during 30 minutes and washed in PBS. The sections were incubated with each lectin, according to the dilution (see Table 1), in a humid chamber overnight at 4°C. Then, the next day, each sample was washed in PBS, and about 50 µL of the streptavidin–peroxidase solution was applied to each sample (Streptavidin Peroxidase Horseradish Vector Labs^®^). The slides were placed in a humid chamber and allowed to rest for 30 min at room temperature. The samples were washed again in PBS and then marked with diaminobenzidine (DAB), watching the marking under a microscope and recording the time of marking. In contrast, the slides were immersed in Harris hematoxylin for 5 s, washed in running water, and then dehydrated in ethanol of increasing concentrations and in the xylene substitute (Neo Clear ^®^). At the end of the procedure, mounting was performed with Canada balsam using cover slips (Acuña et al., 2020).

For the negative control, sections of the same samples were used, where each lectin was replaced by PBS. The positive control was a mouse intestine sample incubated with lectins (López C et al., 2014).

Image analysis was performed using a Leica DM 750 microscope with an integrated digital camera ICC50 W and Leica Microsystems LAS 4.12 software. Marking intensity was classified on a qualitative scale (- negative; + weak; ++ moderate; +++ strong) and were described with digital images. The assessment of the intensity was based on estimates from three trained independent researchers blinded to the samples being observed (Acuña et al., 2020).

## 3 Results

### 3.1 General histological structure

**FIGURE 1:**
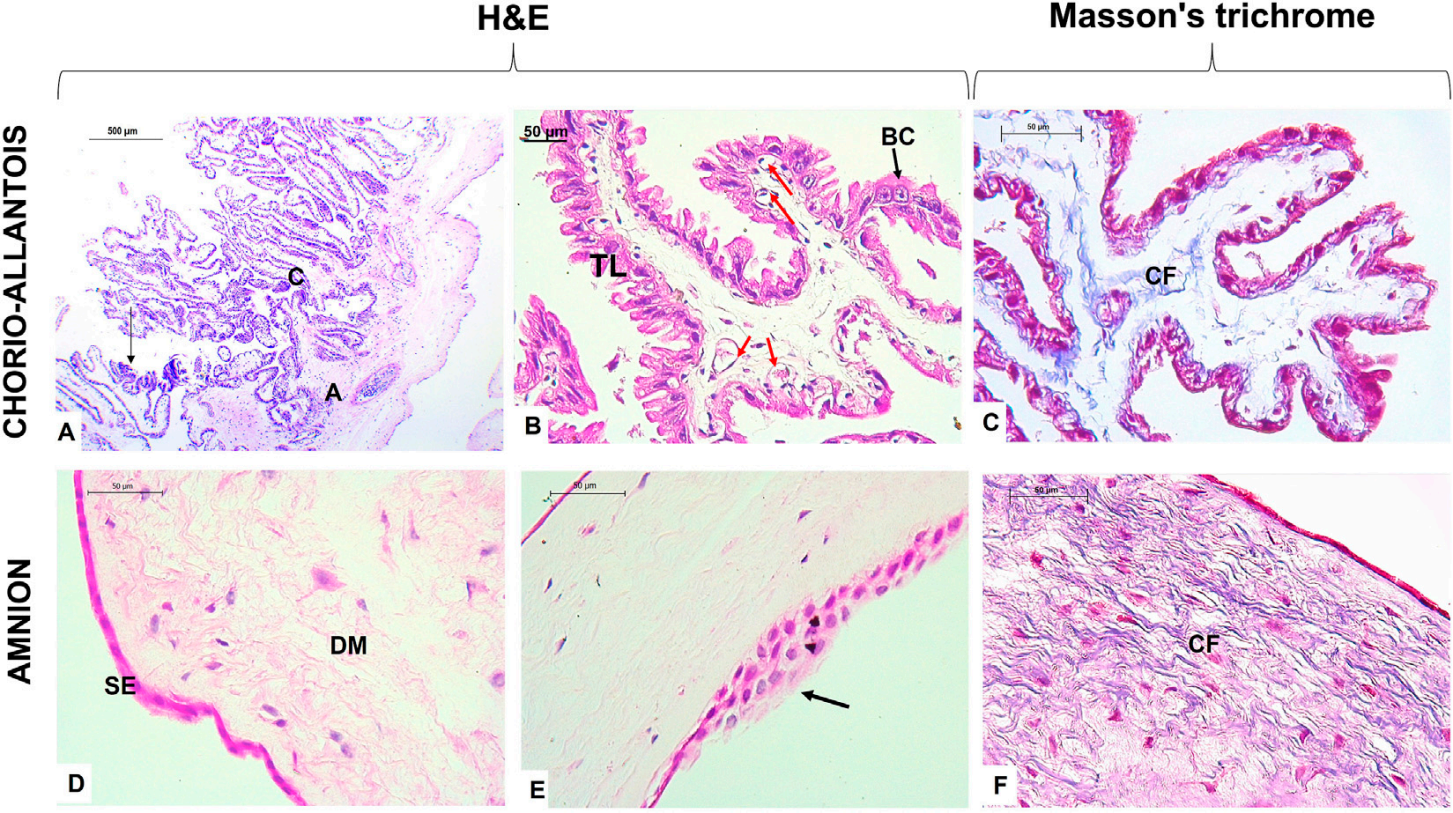
Histological characteristics of the term placenta in alpaca. **(A)** Note the union of the chorion **(C)** and allantois **(A)**, typical of chorio-allantois placentas; the arrow indicates the presence of giant trophoblast cells. **(B)** The chorion presents with villi lined with the trophoblastic layer (TL), and binucleated cells (BC) appear among the columnar cells. In addition, subepithelial blood capillaries (red arrows) are characterized by the epitheliochorial placenta. **(C)** Collagen fibers (CFs) make up the mesenchyme of the chorio-allantois. **(D)** Alpaca term placental amnion membrane demonstrates a simple flat epithelium (SE) layer and dense avascular mesenchyme (DM) with the presence of many fibroblasts. **(E)** Presence of areas with several layers of simple epithelium in the amnion.**(F)** Note the epithelium and fibroblasts stained in intense pink, the collagen fibers (CF) in blue, and the reticular fibers in pink. Scale bar 500 μm **(A)** and 50 μm **(B–F)**.

The term alpaca placenta presented a diffuse epitheliochorial type, characterized histologically by maintaining the chorion adhered to the allantois with a simple cuboidal trophoblast epithelium, where some giant cells at the apical end of the villi were present. A subepithelial layer of fetal blood capillaries was evident. Likewise, the amnion membrane presented a flat epithelium but with some zones of epithelial multilayers and a dense mesenchyme rich in collagen fibers (Figure 1).

### 3.2 Lectin histochemistry

The expression of the glycoconjugates in the chorio-allantoic and amnion membranes of the term alpaca placenta was qualitatively evaluated according to Table 2.

**TABLE 2:** Evaluation of lectin expression in the term alpaca placenta.

Lectin	Scale	Chorio-allantois-marking appointment place	Scale	Amnion-marking appointment place
CON- A	++/+++	Trophoblastic cell cytoplasm	++/+++	Cytoplasm of epithelial cells and mesenchyme
SBA	++	Glycocalyx surface of trophoblastic cells	++/+++	Cytoplasm of epithelial cells and mesenchyme
WGA	+++	Trophoblastic cell cytoplasm	++/+++	Cytoplasm of epithelial cells and mesenchyme
DBA	+	Trophoblastic cell cytoplasm	+	Cytoplasm of epithelial cells and mesenchyme
UEA-I	−/+	Trophoblastic cell cytoplasm	+	Cytoplasm of epithelial cells
RCA-I	+++	Trophoblastic cell cytoplasm	++/+++	Cytoplasm of epithelial cells and mesenchyme
PNA	+/++	Glycocalyx surface of trophoblastic cells	+/++	Glycocalyx surface of epithelial cells
GSL-I	++	Trophoblastic cell cytoplasm	+/++	Cytoplasm of epithelial cells
PSA	+++	Cytoplasm of trophoblastic cells and the endothelium of mesenchymal blood vessels	+++	Cytoplasm of epithelial cells and mesenchyme
LCA	++/+++	Trophoblastic cell cytoplasm	++/+++	Mesenchyme
PHA-E	++/+++	Endothelium of chorion-allantoic blood vessels	++	Cytoplasm of epithelial cells and mesenchyme
PHA-L	++/+++	Endothelium of chorion-allantoic blood vessels	+	Cytoplasm of epithelial cells and mesenchyme
sWGA	++	Glycocalyx surface of trophoblastic cells	++	Cytoplasm of epithelial cells

**FIGURE 2:**
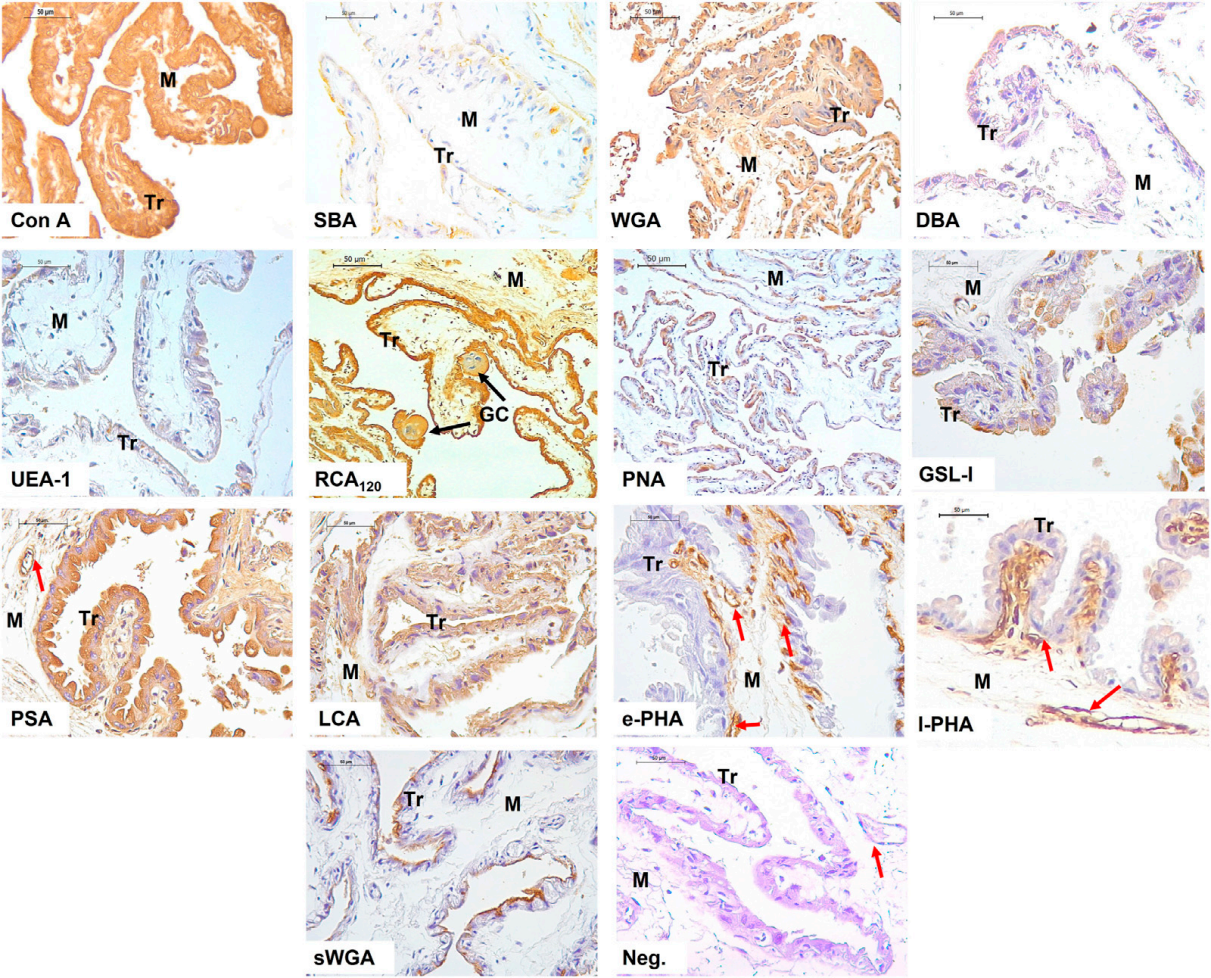
Lectin labeling in the chorio-allantois of the term alpaca placenta. The trophoblastic (Tr) cell cytoplasm strongly labeled for Con A, WGA, RCA_120_, PSA, and LCA lectins. This included labeling of multinucleated giant cells (GC). Labeling with DBA and UEA I lectins was weak. The trophoblast surface glycocalyx stained moderately for SBA, PNA, and sWGA lectins. The rich vasculature (red arrows) trophoblastic subepithelial strongly labeled with the e-PHA and l-PHA lectins representing complex structures, whereas the mesenchymal blood vessels strongly labeled with the PSA lectin. Negative control showed no tissue labeling. Scale bar 50 μm.

**FIGURE 3:**
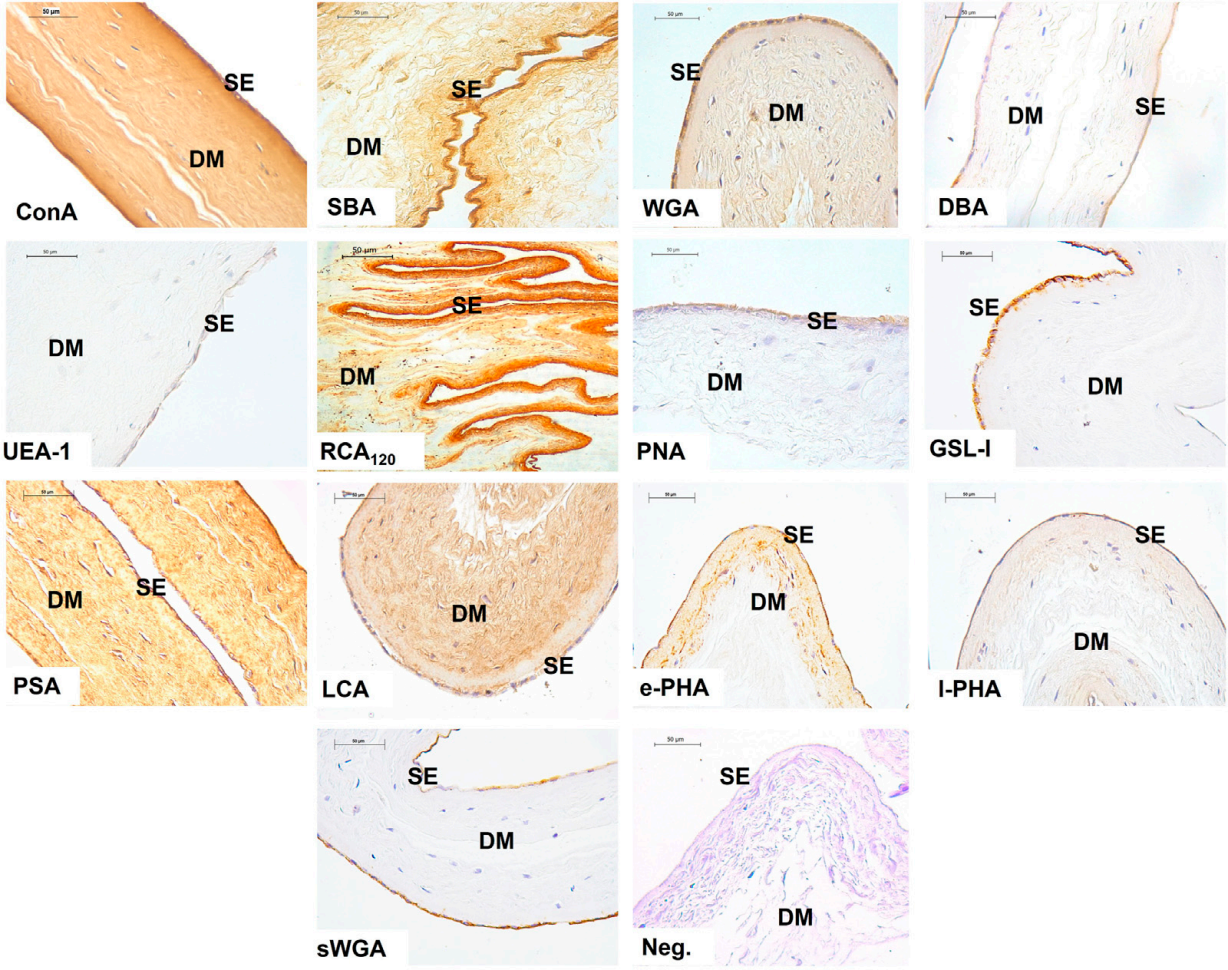
Labeling of lectins in the amnion of the term alpaca placenta. The simple epithelium (SE) and the dense mesenchyma (DM) of the amnion presented strong labeling to lectins Con A, SBA, WGA, RCA_120_, and PSA; and moderate labeling with PNA, GSL I, e-PHA, and sWGA lectins. The latter did not label the mesenchyme. It was weak for DBA, UEA I, and l-PHA lectins. LCA lectin moderately labeled only the mesenchyme. Negative control showed no tissue labeling. Scale bar 50 μm.

The results show the affinity of the chorion-allantoic and amnion membranes of the term alpaca placenta for lectins that refers to structural carbohydrate components of galactose, glucose, mannose, and sialic acid, as well as *N-acetylgalactosamine* and *N-acetylglucosamine*, although they present a low affinity for fucose. Fetal blood capillaries showed affinity for bi- and tri-antennary complex structures and mannose (Figures 2, 3).

## 4 Discussion

The maternal–fetal barrier of some viviparous species has been described to have its own glycocode (Jones et al., 1997), and they are able to modify glycan patterns during the early stage of development, including conception and placental development (Jones et al., 1995). During embryonic and placental development, the glycosylation profile can be modified, particularly by several glycosyltransferases that are regulated by environmental stimuli (Jones et al., 2000; Russo, 2011). Owing to this, different glycan profiles are related to embryonic attachment, control, and inhibition of the trophoblastic invasive process, immunological protection, and the presence of signaling molecules such as hormones, enzymes, and growth factors (Dantzer and Leiser, 1993; Chen et al., 2016). The epitheliochorial placenta presents some interdigitated microvilli in contact with the uterus, which may represent an advanced evolutionary adaptation (Mess and Carter, 2007).

Likewise in other species, the South American camelid placentas demonstrate morphological and functional adaptations to guarantee pregnancy success at high altitudes and low-oxygen levels (Miranda-de la Lama and Villarroel, 2006; Gonzalez-Candia et al., 2021). Some studies already demonstrated that such adaptations perform a type of compensation for the environmental hypoxia, allowing the normal fetal growth in these animal populations (Wilsterman and Cheviron, 2021). Sheep submitted to high altitudes presented an increase on the maternal–fetal contact surface (Parraguez et al., 2006), while in humans, an increase in the lumen of uterine arterial branches enhances the blood flow to the fetus (Moore et al., 2011; Zhao et al., 2021; Dolma et al., 2022).

In this context, in a hypoxic environment, the activated hypoxia-inducible factor (HIF) pathway is able to modulate placental angiogenesis through VEGF and VEGFR1 gene upregulation, increasing the development of new blood vessels and, thus, supplying an efficient blood flow to the conceptus (Macklin et al., 2017). In addition, in hemochorial placentas, the activated HIF pathway also contributes to vascular mimicry, which takes part in fetal blood supply (Macklin et al., 2017). This mechanism may be related to the adaptation of placentas exposed to high altitudes.

Placental angiogenesis is also modulated by glycans presented on the trophoblastic surface, once some galectins, such as Gal-1, are able to modulate vascular signaling, glycosylating the VEGFR receptor, for example, triggering the angiogenic stimulation, even in the absence of its canonical ligand (Cerliani et al., 2017). Under hypoxic conditions, endothelial cells from placental vessels have their glycosylation pattern altered, replacing some sort of glycans, which may stimulate angiogenesis (Cerliani et al., 2017). For these reasons, the understanding of the glycosylation profile of alpaca placentas may elucidate some aspects of its environmental adaptation.

Glycosylation studies on the maternal–fetal interface in camelids pointed differences between uterine and trophoblastic tissue glycosylation (Jones et al., 2000; 2002; Aplin and Jones, 2012). The glycopattern plays an essential role on implantation and pregnancy maintenance, once it acts on the pre-zygotic processes such as spermatozoa selection, implantation, and fetal recognition (Tumova et al., 2021). The present investigation showed the structural conformation of carbohydrates in the alpaca term placenta, immediately after labor, including the chorion-allantois and the amniotic membranes, and also demonstrated the different patterns between them (Figures 2, 3).

Strong labeling for RCA_120_, Con A, and WGA lectins in the chorionic villi trophoblastic cells indicates the presence of glycosidic residues galactose and β-*N-acetylgalactosamine*, glucose and mannose residues, β-*N-acetylglucosamine* and sialic acid residues, respectively. This intense labeling was also evident in the amnion epithelial layer and mesenchyme (Figures 2, 3).

Other investigations on camels’ placentas and South American camelid placentas indicated that the alpaca is the only species among camelids presenting sialic acid in the trophoblast (Jones et al., 2008). We confirmed this result using the WGA lectin, which can bind both sialic acid and *N-acetylglucosamine* oligomers.

As sialic acid and *N-acetylglucosamine* oligomers are also strongly present in humans, moderately observed in armadillos and guinea pigs (Jones et al., 2007), and observed in chorionic villi of porcine placentas at different gestational stages (Sanchis et al., 2009a), it may be inferred that epitheliochorial placentas of pigs and camelids share similar patterns.

WGA lectin is expressed in swine, equine, bovine, ovine, and human placentas; however, its staining was more evident in equine and human trophoblastic cells (Jones et al., 1997; 2017). Among camelids, the guanaco showed more evident WGA labeling in the trophoblast (Jones et al., 2008). sWGA was moderately stained in the glycocalyx of trophoblastic cells and in the epithelial cells of the amnion. Tatsuzuki et al. (2009) observed that cells localized in the brush border of the syncytiotrophoblastic layer of the human placenta have a strong expression of GlcNAc through WGA and sWGA lectins. In the human trophoblastic basement membrane, the expressed mannose (positive by Con A, LCA) and galactose (strongly positive by ECL, RCA_120_) were evident; however, they did not react with lectins from the GlcNAc group or the fucose-lectin group. Finally, Acuña et al. (2023) indicated that WGA positivity is the ancestral condition for Placentalia.

Although camel placentas showed less glycoconjugate reaction when compared to the alpaca, llama, and guanaco, using lectins with Con A, PSA, DBA, and DSA, they presented a greater reaction for SBA lectins (Jones et al., 2008).

The term alpaca placenta showed a strong expression of glucose and mannose through the Con A lectin. Studies carried out in porcine placentas demonstrated the most evident staining of the Con A lectin in blood vessels (Sanchis Gabriela and Chanique Analía, 2012). This result was also evident in other placental types, including the maternal and fetal capillaries of equine placentas (Jones et al., 1999).

Glycosylated residues of α-mannose and α-glucose that bind to Con A are presented in the placental vessel endothelium in swine and a coinciding result with that found in human placentas from normal pregnancy, demonstrating mild expression of α-mannose in placental vessels (Jones et al., 1997). Con A positivity is the plesiomorphic condition for Placentalia and is preserved in all species (Acuña et al., 2023).

Fucose–carbohydrate weak expression was also described using the UEA I lectin, highlighted in the chorion and amnion in the term alpaca placenta. Similar result was also found in camels (Aplin and Jones, 2012). The staining of this lectin in the alpaca placenta was not evident in the studies carried out by Jones et al. (2008). In another study, UEA I presence was found in the placenta in 145, 170, and 350 days of gestation in camels and occasionally on day 283 in alpaca (Jones et al., 2002). Recent investigations carried out in the alpaca uterus during superovulatory periods and normal treatment did not demonstrate any labeling of this lectin (López C et al., 2014). In the equine placenta, there was no evidence of fucose staining (Jones et al., 1999), which was not observed in the microvillus membrane of the human placenta as well (Jones et al., 1997). Such findings indicate changes in fucosylation in the alpaca term placenta. Aplin and Jones (2012) indicated that the conservation or change in the expression of the fucosyltransferase gene over evolutionary time plays a role in the stability at the maternal–fetal interface.

The α-*N-acetylgalactosamine* presence was evidenced by SBA and DBA lectins in the term alpaca placenta; however, staining was different. In both the chorion and amnion, SBA lectin staining was moderate, while it was very weak with DBA lectin. Studies carried out in camel and alpaca placentas demonstrate that no binding to the DBA lectin was found (Jones et al., 2002). However, in alpaca, it was possible to find glycosyltransferase activity on the cell surface of chorionic microvilli but a clear decrease with advancing gestation. According to Jones et al. (2002), the DBA lectin did not stain camel trophoblasts, but occasional granules were evident in horse and donkey trophoblasts. The DBA lectin shows differentiated labeling in placentas of different species and is strongly stained in ovine and bovine trophoblasts, moderately expressed in horses, but absent in pigs and humans (Jones et al., 2007). However, SBA lectin is stained in sheep, cattle, horses, and pigs and is not seen in humans (Jones et al., 1997).

Our results showed the presence of galactose through the PNA lectin with a moderate labeling in the glycocalyx of the chorionic cells of the term alpaca placenta. Moreover, the GSL I lectin marked the cytoplasm of the trophoblastic cells. In the amnion, it was moderate in the glycocalyx (PNA) and epithelial cell cytoplasm (GSL I), and weak in mesenchyme.

Studies highlight the presence of galactosylated PNA-binding residues in endometrial glands at the end of pregnancy. Thus, *ß-Gal* (1,3) GalNAc residues have been reported in the endotheliochorial dog placentas, specifically in the glandular cells of the placenta close to labor (Fernández et al., 2000), likewise in cat trophoblasts (Fernández et al., 2014). In contrast, in first-trimester human placental tissues, the AHA lectin, which shares specificity with the PNA lectin, was shown to be strongly associated to glandular secretions and the glycocalyx of the glandular epithelium (Jones et al., 2010).

Mannose presence with the PSA and LCA lectins was well-characterized in the trophoblastic cell cytoplasm, showing granules. Likewise, it strongly stained the endothelium of fetal blood capillaries, and the amniotic epithelium and mesenchyme, suggesting the importance of mannose as a kind of sugar in the term alpaca placenta. Studies with this lectin indicate that its expression is present in all species but in a variable manner. Regarding the human term placenta, the trophoblastic cytoplasm is strongly stained, along with the basement membrane and endothelial membrane (Tatsuzuki et al., 2009). However, it is poorly characterized and expressed in equine, porcine, and mink trophoblasts and occasionally verified in cattle and sheep, strongly marking the fetal blood capillaries among all these species (Jones et al., 1997).

l-PHA lectin staining, which has non-bisected tri-antennary complex structures, was evident in fetal blood capillaries from the term alpaca placental chorion and weak granule-shaped staining in trophoblast cytoplasm, as reported in sheep and pigs by Jones et al. (1997). Nevertheless, in horses, it had a slight staining in fetal blood capillaries, trophoblasts, and the endothelium (Jones et al., 1999). On the other hand, in cattle, blood capillaries and giant cells were moderately stained, while in humans, there was moderate staining in trophoblasts and mild staining in fetal blood capillaries (Jones et al., 1997). Camels did not show marking of this lectin in the trophoblast in the term placenta like in alpaca (Jones et al., 2000). In the amnion of the alpaca, the l-PHA lectin is slightly marked in the cytoplasm of the epithelial cells and the mesenchyme.

The e-PHA lectin expresses complex bisected bi/tri-antennary structure complex, stained strongly like l-PHA, and the fetal blood capillaries of the chorion, but did not stain the trophoblast. This is a different result from that found in camels, which presented mild positive staining in the trophoblast. In horses and donkeys, it demonstrated moderate staining in the trophoblast (Jones et al., 2000). Sanchis et al. (2009b) found abundant staining of e-PHA-binding oligosaccharides in the chorion villi in term porcine placentas, which coincided with the strong staining intensity found at the swine maternal–fetal interface in a study carried out by Jones et al. (2004). Similar results were described in peccary and camel placentas (Jones et al., 2000; 2002; 2004).

When Jones et al. (2008) compared the staining in placentas of several camelids, no positive staining for e-PHA or l-PHA lectins was found at the maternal–fetal interface. This result differs from our study in alpacas, in which a very strong labeling was present in fetal blood capillaries, suggesting the importance of this e-PHA lectin during labor in the mentioned species. e-PHA positivity is the ancestral condition for Placentalia and is preserved in species except for *Lama glama* (Acuña et al., 2023).

The structural diversity of the maternal–fetal interface is accompanied by a very high degree of biochemical diversity in glycans, as evidenced by the widely differing patterns of lectin binding in each layer of the placenta among species (Majewska et al., 2013). This difference in the glycosylation profile is more evident in chorionic cells, varying during different gestational stages in camelids, such as alpaca and camels, other domestic animals, and in humans (Majewska et al., 2011). The expression of glycosidic residues shows the evolutionary history of the placenta among eutherians and the phylogenetic relationships between species of different taxa (Acuña et al., 2023).

Studies carried out at the maternal–fetal interface in camelids (Jones et al., 2008) confirm that the greatest difference in glycan expression occurs at the level of the fetal trophoblast and not in the maternal uterus, particularly for hybridization purposes among different species of camelids (Jones et al., 2002). Our results corroborate the aforementioned study that there is a greater variation in the carbohydrate pattern in the trophoblast, which varies due to external factors. Regarding alpacas, the decisive environmental condition is their natural habitat, where oxygen pressure levels are low due to high altitudes, which is enhanced by their natural hyperglycemic profile (Cebra et al., 2001).

This suggests that further studies on the role of carbohydrates in several physiological actions in animals that survive at high altitudes are required. The possible major role of sugar chains in the placenta may involve the exchange and metabolism of various substances such as minerals, production of a number of hormones, and the placental barrier (Tatsuzuki et al., 2009).

## 5 Conclusion

In conclusion, our study described the term alpaca placenta glycosylation profile, which highlighted the heterogeneous distribution of carbohydrate residues along the trophoblast, amniotic epithelium, mesenchyme, and fetal blood capillaries. These glycocodes may modulate some pro-angiogenic activities in the alpaca placenta, contributing to its adaptation to high altitudes and low oxygen levels, which guarantees the pregnancy maintenance and normal fetal growth.

## Data Availability

The original contributions presented in the study are included in the article/supplementary material; further inquiries can be directed to the corresponding author.
